# Alteration of the lysophosphatidic acid and its precursor lysophosphatidylcholine levels in spinal cord stenosis: A study using a rat cauda equina compression model

**DOI:** 10.1038/s41598-019-52999-5

**Published:** 2019-11-12

**Authors:** Baasanjav Uranbileg, Nobuko Ito, Makoto Kurano, Daisuke Saigusa, Ritsumi Saito, Akira Uruno, Kuniyuki Kano, Hitoshi Ikeda, Yoshitsugu Yamada, Masahiko Sumitani, Miho Sekiguchi, Junken Aoki, Yutaka Yatomi

**Affiliations:** 1 Department of Clinical Laboratory Medicine, The University of Tokyo, Tokyo, Japan; 2 Department of Anesthesiology and Pain Relief Center, The University of Tokyo, Tokyo, Japan; 3 Department of Integrative Genomics, Tohoku Medical Megabank Organization, Miyagi, Japan; 4 Medical Biochemistry, Tohoku University School of Medicine, Sendai, Miyagi Japan; 5 Laboratory of Molecular and Cellular Biochemistry, Graduate School of Pharmaceutical Sciences, Tohoku University, Miyagi, Japan; 6 Department of Pain and Palliative Medicine, The University of Tokyo Hospital, Tokyo, Japan; 7 Department of Orthopaedic Surgery, Fukushima Medical University, School of Medicine, Fukushima, Japan

**Keywords:** Lipidomics, Neuropathic pain

## Abstract

Cauda equina compression (CEC) is a major cause of neurogenic claudication and progresses to neuropathic pain (NP). A lipid mediator, lysophosphatidic acid (LPA), is known to induce NP via the LPA_1_ receptor. To know a possible mechanism of LPA production in neurogenic claudication, we determined the levels of LPA, lysophosphatidylcholine (LPC) and LPA-producing enzyme autotaxin (ATX), in the cerebrospinal fluid (CSF) and spinal cord (SC) using a CEC as a possible model of neurogenic claudication. Using silicon blocks within the lumbar epidural space, we developed a CEC model in rats with motor dysfunction. LPC and LPA levels in the CSF were significantly increased from day 1. Importantly, specific LPA species (16:0, 18:2, 20:4) were upregulated, which have been shown to produce by ATX detected in the CSF, without changes on its level. In SC, the LPC and LPA levels did not change, but mass spectrometry imaging analysis revealed that LPC was present in a region where the silicon blocks were inserted. These results propose a model for LPA production in SC and CSF upon neurogenic claudication that LPC produced locally by tissue damages is converted to LPA by ATX, which then leak out into the CSF.

## Introduction

Lumbar spinal canal stenosis (LSS) is the narrowing of the spinal canal and is characterized by the presence of low back pain and radiating leg pain, including cramping muscle pain and tired legs. All of these painful symptoms are associated with symptoms of neuropathic pain (NP). These become prominent and worse with ambulation, but most are relieved at rest. In close relation with neuropathic pain symptoms with ambulation, LSS is characterized as a reduced walking distance and short intermittent sensorimotor deficits, which are called as neurogenic claudication. Again, neurogenic claudication is unremarkable at rest, but becomes prominent and worse during walking. Such neurogenic signs and symptoms of LSS generally depend on its anatomical grade and are mostly triggered by exercise^1^.

LSS is the most common reason for lumbar spine surgery, especially in older people, and the prevalence of LSS has increased in recent years because of the aging of the population and the improved quality of imaging studies^2^. Long-lasting LSS leads to irreversible nerve damage of the cauda equina and the development and maintenance of neurogenic claudication and NP, which affects millions of people worldwide^3^.

NP is defined by the International Association for the Study of Pain (IASP) as pain caused by a lesion or disease of the somatosensory nervous system, and NP is characterized by abnormal pain symptoms such as hyperalgesia, hypoesthesia and allodynia^4,5^. In contrast to the situation in LSS, hypoesthesia and allodynia at rest are certainly common signs and symptoms of other peripheral neuropathies. But, painful motor impairment of LSS with ambulation is also considered in reference to neuropathic pain, because gabapentinoids, typical analgesics for NP, have empirically demonstrated improvement in both pain and walking distance^1,6–10^.

The underlying pathophysiology of NP, potentially causing neurogenic claudication, is not well defined, and some theories regarding the mechanism of NP have been proposed using animal models^11–14^. Several reports have indicated the importance of lysophosphatidic acid (LPA, 1- or 2-acyl-sn-glycerol 3-phosphate) in the development and maintenance of NP. In our recent human study, increasing LPA of the cerebrospinal fluids (CSF) was linearly associated with severity of neurogenic claudication and the anatomical narrowing grade of LSS^15^. LPA is a lysophospholipid (LPL) and acts as a potent lipid mediator through defined G protein-coupled receptors (GPCRs). LPA is produced from lysophosphatidylcholine (LPC) by the hydrolyzing action of autotaxin-lysophospholipase D and from phosphatidic acid (PA) by the action of phospholipase A (PLA)-type enzymes^16^. LPA performs a wide variety of biological activities, including cellular proliferation, the prevention of apoptosis, cell migration, cytokine and chemokine secretion, platelet aggregation and neurite retraction^17–20^. Regarding pain, LPA activates ion channels such as TRPV1^21^ in peripheral nociceptor endings directly and indirectly by inducing the release of histamine from peripheral immunopotent cells such as leukocytes and macrophages, leading to nociceptive pain through its receptor LPA_1_^22,23^. Furthermore, LPA initiates and maintains NP^13,24,25^; for example, the intrathecal injection of the LPA imitates behaviors of NP following nerve ligation. In an animal model of spinal cord injury (SCI), increased levels of LPA together with the induction of microglia/macrophages and demyelination have been observed in the injured spinal cord tissue^26^. Very recently, we were the first, to our knowledge, to report that LPA levels in the CSF are significantly correlated with the clinical symptoms of patients with different etiologies and intensity of NP^27^. Also, we confirmed that the levels of LPA, LPC were higher in the CSF in patients with LSS and the levels of LPC species were correlated with those of corresponding species of LPA^15^. Enhancement of the LPC level during NP is still remaining unclear, but for the increased level of the LPA in CSF could be explained from LPC by hydrolyzing action of the ATX that already exits in CSF.

Most previous basic studies have measured LPA levels in damaged spinal cord tissues subsequent to anatomical damage, such as peripheral nerve ligation, or chemical injury, such as the injection of substances, including LPA, both of which cause severe demyelination and axonal damage^26,28^. In the present study, we used a model of cauda equina compression (CEC), which does not directly injure nervous tissues but can lead to neurogenic claudication associated with NP (i.e., low back pain and radiating leg pain)^29^. The CEC model is known to be a preclinical model of lumbar spinal canal stenosis (LSS). Focusing on our recent finding that LSS patients with severe neurogenic claudication demonstrate more increasing levels of LPA in cerebrospinal fluids than those with mild neurogenic claudication^15^, we aimed to determine the levels of LPC and LPA in the cerebrospinal fluid (CSF), plasma and spinal cord of a rat model of CEC by measuring LPA receptor expression and the spatiotemporal alteration of LPC concentration in the spinal cord by means of matrix-assisted laser desorption/ionization mass spectrometry imaging (MALDI-MSI) technology.

## Results

### Development of a lumbar spinal stenosis model (CEC) for the generation of neurogenic claudication associated with NP

**Figure 1:**
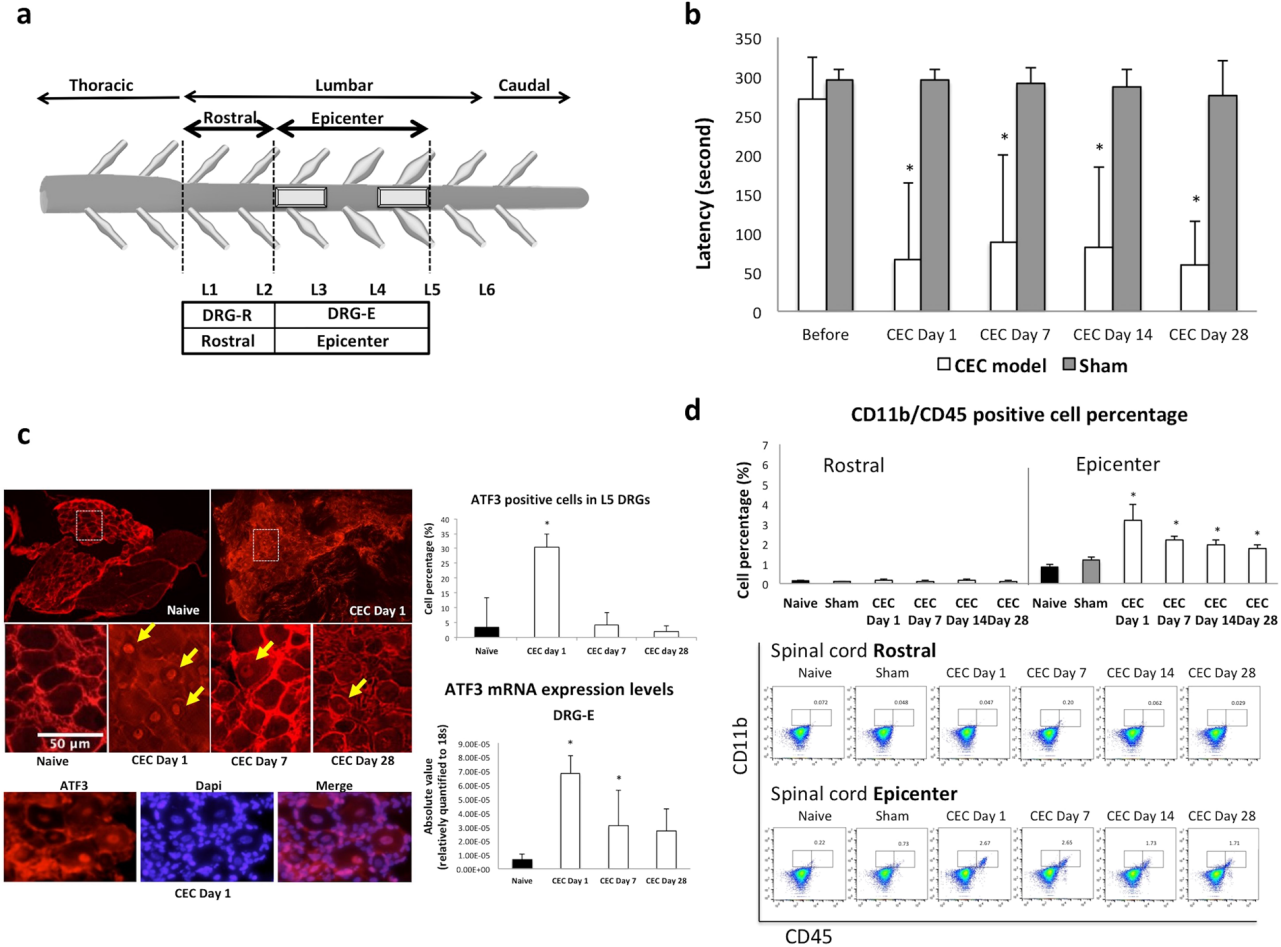
Establishment of a rat model of LSS or CEC. (**a**) A schematic drawing of the rat spinal cord, the location of the inserted silicon blocks (the L3 and L5 epidural space) and the collection of tissue for further analysis. (**b**) Rotarod was used to test motor function before surgery and on days 1, 7, 14 and 28 after surgery. Compared to that in the sham-operated rats, the duration of running time was significantly (*p < 0.05) decreased in the CEC model rats beginning on the first day after surgery. (**c**) A representative image of IHC staining of the L5 DRG after the placement of the 2 silicon blocks with an ATF3 antibody showing the strong expression of ATF3 (arrowheads) in the nuclei (400×). Upper 2 images (40×) show the origin of the zoom-in DRGs. Dapi staining (lower) for co-localization of the ATF3 stained nucleus. The ATF3-positive cells on both sides of the L5 DRG were counted, and the calculated percentage of positive cells is shown (n = 5). ATF3 was most increased in the CEC rats on day 1 after surgery. On day 7 and day 14, only a few nuclei were labeled with ATF3 in the CEC rats. ATF3 mRNA expression levels in the DRGs at the epicenter (L3, L4 and L5) were also compared for each time point (n = 5). The percentage of ATF3-positive cells and the ATF3 mRNA expression were significantly (*p < 0.05) increased in the CEC rats on day 1. (**d**) Flow cytometry analysis showed the infiltration of CD11b/CD45 double-positive cells into the spinal cord after surgery. As a control, we used tissues from the same regions from naive and sham-operated rats. The results from the rostral segments (above the silicon blocks) and the epicenter segments (where the 2 silicon blocks were inserted) of the spinal cord tissue from the CEC rats on days 1, 7, 14 and 28 are shown. The percentage of CD11b/CD45 double-positive cells (upper panel) in the epicenter segments was markedly (*p < 0.05) enhanced, indicating the infiltration of macrophages and microglia into the site of the injury. Representative forward/scatter dot plots showing the percentage of CD11b/CD45 double-positive cells (lower panel).

Two silicon blocks (4 mm × 1 mm × 1 mm) were placed in the L3 and L5 epidural space under surgical microscopy as described in the Materials and Methods section. The experimental protocols for the development of this CEC model of NP and for tissue sample preparation for further analyses are shown in Fig. 1a. No wound infection or paralysis was observed after the surgical procedure. We successfully established the CEC model and validated it by demonstrating consistent motor deficiencies using the rotarod test at selected time points (Fig. 1b). In comparison with that in the sham-operated rats, walking ability was significantly impaired in the CEC model rats soon after surgery (days 1, 7, 14 and 28).

Next, we performed IHC analysis of activating transcription factor 3 (ATF3) expression in the dorsal root ganglion (DRG) at spinal level L5, to determine if its expression is enhanced in response to spinal cord injury induced by compression by the implanted silicon blocks. ATF3 is a transcription factor that regulates the expression of various proteins and is a biomarker of tissue damage^30^. Figure 1c depicts ATF3 immunohistochemistry in the L5 DRG at days 1, 7 and 28. The number of ATF3-positive cells was increased in the very early stage of CEC (day 1) but was ameliorated at days 7 and 28. We also determined the mRNA expression level of ATF3 in the DRG of the L5 spinal nerve (Fig. 1c). Increased ATF3 mRNA expression was observed in the DRGs at the spinal level of the CEC epicenter segments (L3, L4 and L5) of the CEC model rats at day 1 and then gradually decreased with time, which was similar to the findings obtained by IHC analysis.

We next investigated CD11b and CD45 expression in the spinal cord by flow-cytometry, as CD11b/CD45 double-positive cells represent microglia/macrophage populations. Figure 1d shows the accumulation of CD11b/CD45-positive cells in the site of spinal cord compression by the silicon blocks at the spinal cord, suggesting that CEC can induce indirect injury in the spinal cord and an enhancement of immune cell infiltration into the injured site compared to that in the uncompressed site (i.e., the rostral segments in Fig. 1a).

### Enhanced expression levels of LPA_1_, LPA_5_ and LPA_6_ mRNAs in the CEC model

**Figure 2:**
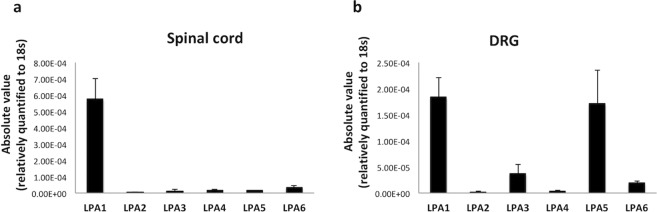
The mRNA levels of LPA receptors in the rat spinal cord and DRGs. To confirm and compare the expression levels of LPA receptors in normal (healthy) tissue samples, we evaluated the spinal cord (**a**) and DRGs (**b**) of the epicenter segments from naive rats. The expression levels are shown as absolute values relative to eukaryotic ribosomal 18s as an internal control. LPA1 was the most abundant receptor in the spinal cord (**a**) and DRGs (**b**), whereas the other LPA receptors (LPA2–6) were expressed at low levels in the spinal cord (**a**). In addition to the levels of LPA1, LPA5 levels were also high in the DRGs and were followed by LPA3 and LPA6 levels (**b**).

LPA, which plays the role of a potent lipid mediator by acting with differing affinities on six LPA receptors (LPA_1-6_), can be detected *in vivo* as a mixture of different fatty acid species (LPA species). First, we measured the mRNA levels of all six LPA receptors in the spinal cord and DRGs of naive rats to determine the basal expression levels (Fig. 2). In the spinal cord tissue samples, LPA_1_ mRNA was the most abundant, followed by LPA_6_, LPA_5_, LPA_4_ and LPA_3_ mRNA. LPA_2_ mRNA was virtually absent.

In the DRG, LPA_1_ and LPA_5_ were detected at high levels, followed by LPA_3_ and LPA_6_. In contrast, LPA_2_ and LPA_4_ mRNA expression levels were very low.

**Figure 3:**
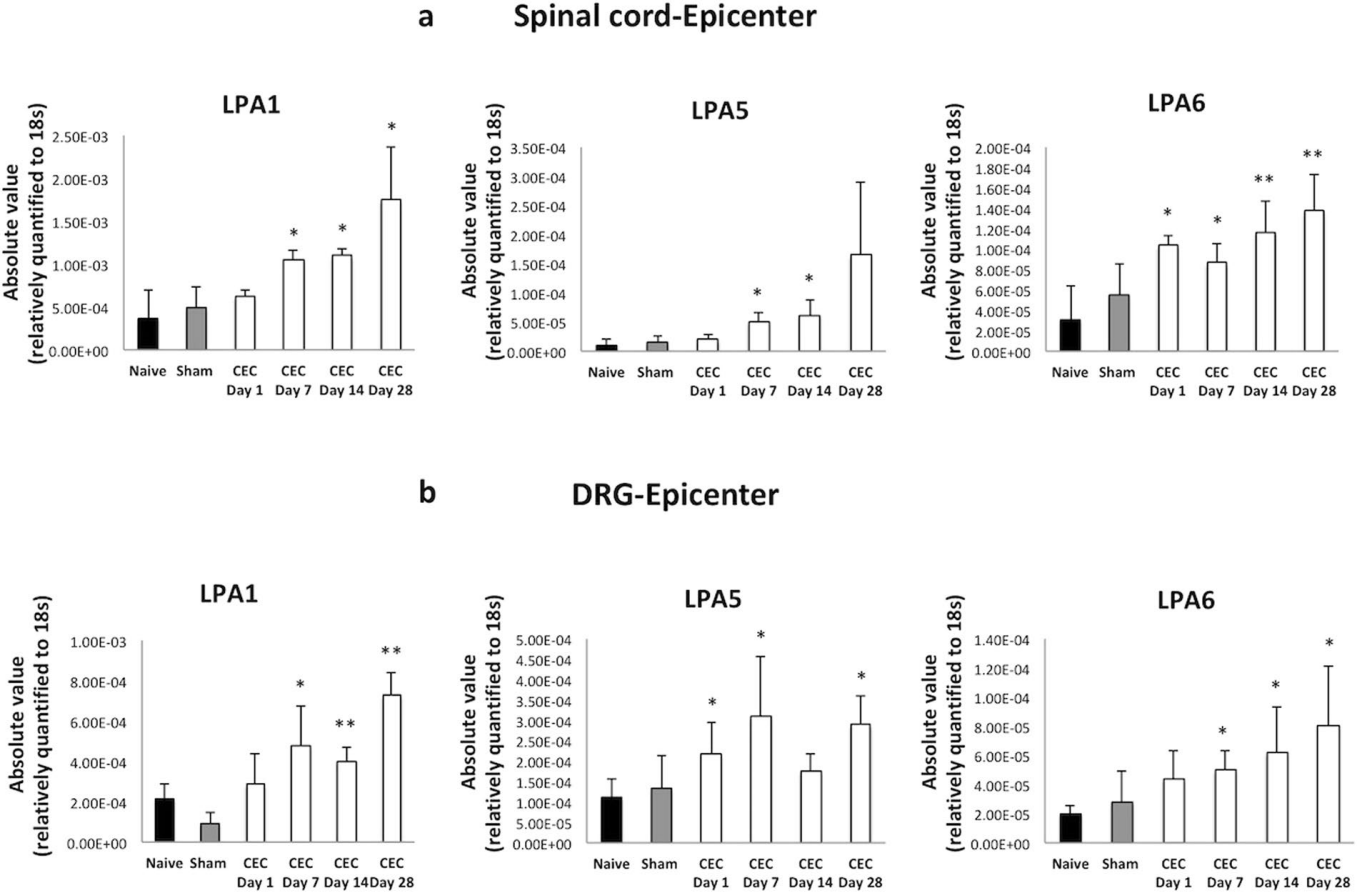
mRNA expression levels of LPA1, LPA5 and LPA6 in the epicenter segments of the spinal cord and the epicenter segment DRGs gradually increased following CEC. (**a**) In the epicenter segments of the spinal cord, LPA1, LPA5 and LPA6 levels significantly (*p < 0.05, **p < 0.01) increased over time in the CEC model group compared to the naïve and sham-operated groups. A similar pattern was observed in the DRGs from the epicenter segments (**b**).

We also determined the mRNA levels of all six LPA receptors in the rats that underwent CEC. However, we did not detect any changes in the mRNA expression of LPA_2_, LPA_3_ and LPA_4_ in either the spinal cord or DRG. Figure 3a,b show the mRNA expression levels of LPA_1_, LPA_5_, and LPA_6_ in the spinal cord and DRGs at the spinal level of the CEC epicenter segments. Here, LPA_1_, LPA_5_ and LPA_6_ mRNA expression was found to be significantly enhanced. The mRNA levels of the three LPA receptors increased gradually over time, peaking at day 28. Furthermore, in the absence of direct compression by silicon blocks, LPA_1_, LPA_5_ and LPA_6_ mRNA levels in the DRGs at the epicenter segments exhibited a gradual enhancement, mimicking those in the spinal cord. In the rostral segments of the CEC model rats, which were not directly compressed by silicon blocks, the levels were immediately enhanced in both the spinal cord and the DRGs compared to those in the naive and sham-operated groups beginning the first day after the surgical procedure (Supplemental Fig. 1).

### LPC and LPA levels are increased in the CSF and plasma of the CEC model group

**Figure 4:**
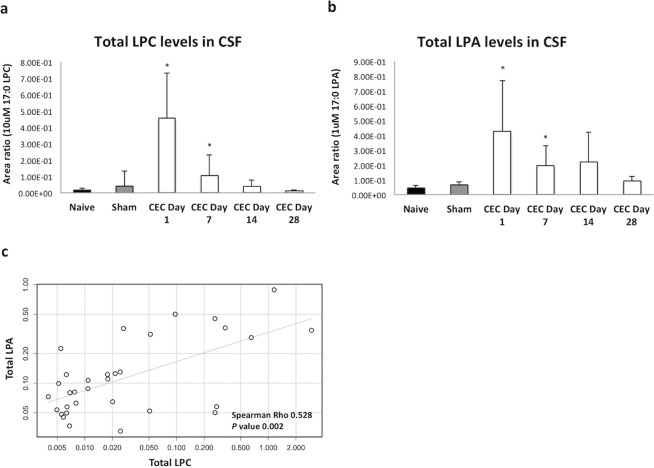
Increased levels of LPC and LPA in the CSF and their correlations. Using LC-MS/MS, LPA and LPC levels were measured in the CSF from the CEC model rats on days 1, 7, 14, 28 and from the naive, sham-operated group rat as a control. The levels of total LPC in the CSF (**a**) and of total LPA in the CSF (**b**) were adjusted to the area ratio of 10 µM 17:0 LPC or 1 µM 17:0 LPA, respectively. Total LPC and total LPA levels were significantly (*p < 0.05) increased in the CSF from the CEC rats on day 1. (**c**) Correlations between LPC levels and LPA levels in the CSF. All of the values measured in (**a**,**b**) were plotted. Nonparametric Spearman’s rank correlation was used, and a strong correlation was observed between the LPC and LPA levels in the CSF (Rho = 0.528, *p*-value = 0.002). Total 4 independent experiments performed for each group.

It is well accepted that LPA species are converted from their corresponding LPC species by autotaxin. In the CSF samples, the levels of both LPC and LPA were under detectable levels in the naive and sham-operated groups. However, the total levels of LPC and LPA in the CSF were significantly (*p < 0.05) increased in the early stages of the CEC (i.e., days 1 and 7) as shown in Fig. 4a,b. None of the LPC and LPA species exhibited significantly increased levels (Supplemental Fig. 2A,B). It is noted here that a few samples showed irregularly high levels of LPC and LPA, most likely due to microhemolysis. Regarding species of the LPC: 14:0, 16:0, 16:1, 18:0, 18:1, 18:2, 18:3, 20:3, 20:4, 20:5, 22:5, 22:6 and species of the LPA: 16:0, 16:1, 18:0, 18:1, 18:2, 18:3, 20:3, 20:4, 20:5, 22:6 were detected and measured in CSF of the CEC. As we confirmed recently^15^ that the levels of LPA, LPC were higher in the CSF in patients with LSS and the levels of LPC species were correlated with the corresponding species of LPA, this time we also checked correlation between species of the LPC and LPA. As shown in Table 1 similar to previous results there were also significant correlations between corresponding species of LPA and LPC, especially 18:2, 20:4 which are known to be hydrolyzed by the action of ATX from LPC^31^.

**Table 1:** Correlation between LPA and LPC species in CSF.

LPA	Spearman Rho	LPC
	*p* value	
18:1	0.352	18:1
	*0.04	
18:2	0.596	18:2
	***0.0003	
18:3	0.478	18:3
	**0.005	
20:3	0.523	20:3
	**0.002	
20:4	0.501	20:4
	**0.003	
20:5	0.557	20:5
	***0.0009	
22:6	0.515	22:6
	**0.002	

We evaluated correlation between the CSF levels of measured LPA species and the corresponding LPC species, (*p < 0.05, **p < 0.01, ***p < 0.001).

In the plasma, LPC species (LPC-14:0, 16:0, 18:1, 18:2, 18:3 and 20:5) were significantly elevated in the CEC model group in comparison to the naive and sham-operated groups (Supplemental Fig. 2C). For LPA, 7 species (LPA-16:0, 18:1, 18:2, 20:3, 20:4, 20:5 and 22:6) were detectable, all of which were significantly increased in the CEC model (Supplemental Fig. 2D).

Next, we determined whether there are correlations between the total level of LPC, and the total level of LPA in the CSF. As shown in Fig. 4c, we observed a strong correlation between the total level of LPC and the total level of LPA in the CSF (Spearman test: Rho = 0.53, p-value = 0.002). No correlations were observed between ATX activity and the total levels of either LPC or LPA (data not shown). These results would suggest that, at first, the levels of LPC in the CSF increase due to nerve compression in the very early stage of the CEC; that, secondarily, physiologically present ATX in the CSF contributes to the increased spontaneous hydrolysis of LPC to corresponding LPA.

### LPA and LPC levels in the spinal cord tissue samples

**Figure 5:**
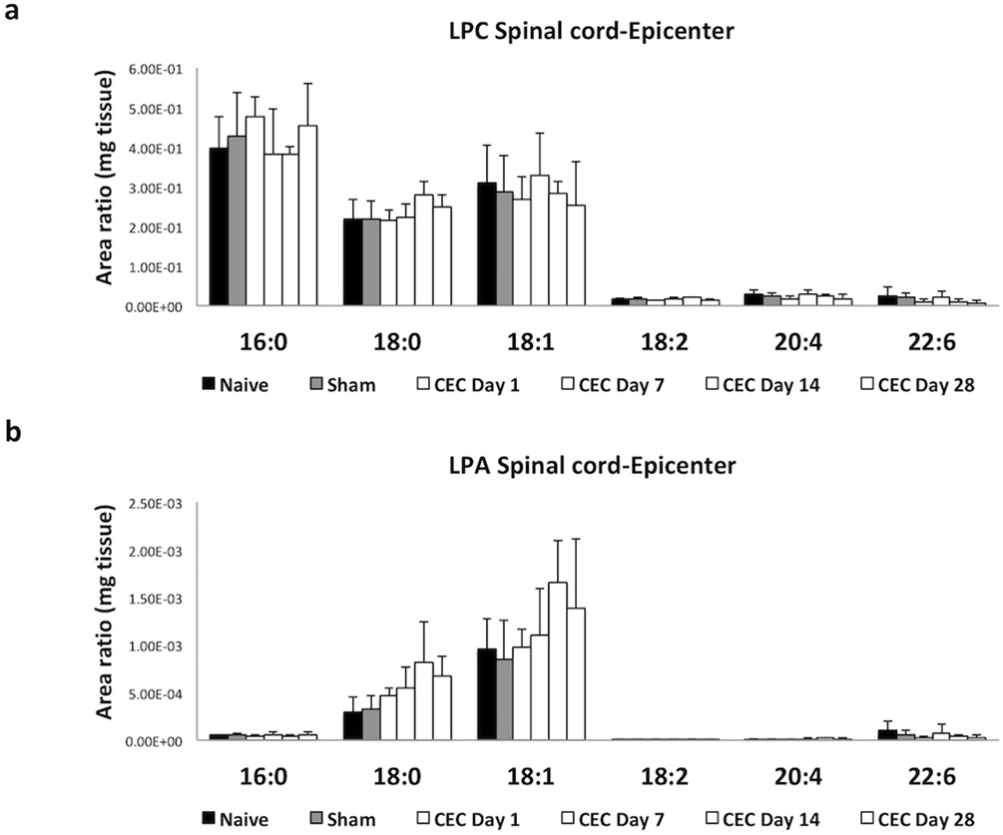
Levels of LPC species and LPA species in the epicenter segments of the spinal cord in the CEC model rats. The levels of LPC species (**a**) and LPA species (**b**) were measured using LC-MS/MS and adjusted to the area ratio of the mass (in mg) of the tissue. There were no significant changes in the levels of LPC and LPA in the spinal cord samples, except for the tendency of the levels of some LPA species to be increased in the CEC model group.

We then determined the levels of LPA and LPC in the spinal cord of the CEC model rats. As described above, the spinal cord tissues were divided into the rostral and epicenter segments. Unlike in the plasma, the levels of LPC in the rostral segments (data not shown) and epicenter segments (Fig. 5a) were not changed compared with those in the tissues from the naive and sham-operated groups. Similarly, we did not detect obvious changes in the level of LPA in the rostral and epicenter segments of the spinal cord in the CEC model rats (data not shown). Again, we observed a slight increase in the level of LPA 18:0 and LPA 18:1 in the epicenter segments of the spinal cord, but these changes were not statistically significant (Fig. 5b). Unlike in CSF, the LPC and LPA levels in SC did not change dramatically.

### MALDI-MSI analysis of LPC in the spinal cord of the CEC model rats

**Figure 6:**
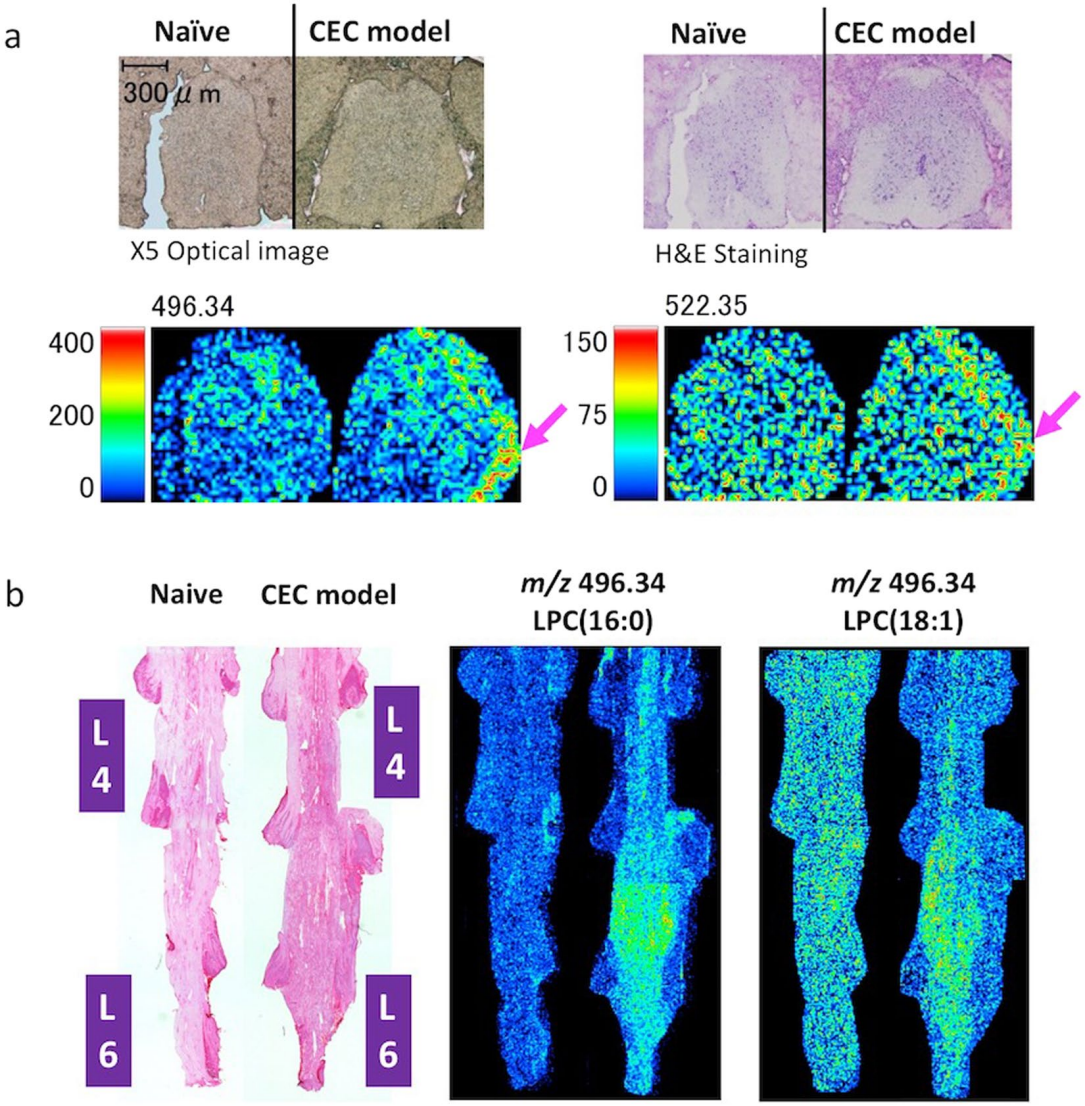
MALDI-MSI analysis of the levels and localization of LPC 16:0 and 18:1 in two different regions of the spinal cord in the CEC model rats. For MALDI-MSI analysis, spinal cord tissues from naive or CEC model rats from day 14 were used. LPC 16:0 and 18:1 were detected by monitoring ions at m/z 496.34 and 522.35, respectively. (**a**) Optical images and hematoxylin-eosin (HE) staining of transverse sections of the spinal cord. The arrowheads indicate the enhanced signals that correspond to LPC 16:0 and LPC 18:1. (**b**) HE staining and MALDI-MSI analysis of coronal sections of the area of the spinal cord where the silicon blocks were placed in naive and CEC model rats. The HE staining images were taken by the microscope (LMD6000, Leica). Enhanced signals were detected in the area where the silicon blocks were placed in the CEC model rats on day 14, indicating increased levels of LPC 16:0 and 18:1.

In general, LC-MS/MS analyses overlook some changes in small regions of tissue. Therefore, we performed MALDI-MSI analysis to determine the distribution of LPC and LPA in transverse and coronal spinal cord tissue slices of the epicenter segments (Fig. 6a,b). LPC 16:0 and LPC 18:1 were detected by monitoring ions at *m/z* 496.34 and 522.35, respectively. Figure 6a shows optical and HE-stained images of the transverse slices together with LPC 16:0 and LPC 18:1. In the transverse slices, the levels of LPC 16:0 and LPC 18:1 were enhanced compared to those in the spinal cord of naive rats, in the local regions where the silicon blocks were inserted (marked by the arrowhead). MALDI-MSI analysis of the coronal slices (Fig. 6b), similar to that of the transverse slices, confirmed the clear enhancement of LPC 16:0 and LPC 18:1 in the epicenter segments where the silicon blocks were located. Although we could not detect the ions derived from LPA by MALDI-MSI.

### Establishment of sources of the increased LPA in CEC model

There are mainly two pathways to synthesize LPA, one is from LPLs such as LPC, LPE, LPG, LPI, and LPS by LysoPLD activity, second is from PA by PA-specific PLA1^16^.

As the first pathway, we showed significantly increased levels of the LPC in CSF and plasma of the CEC model with a correlation between total or corresponding species of the LPA (Fig. 4, Table 1).

**Figure 7:**
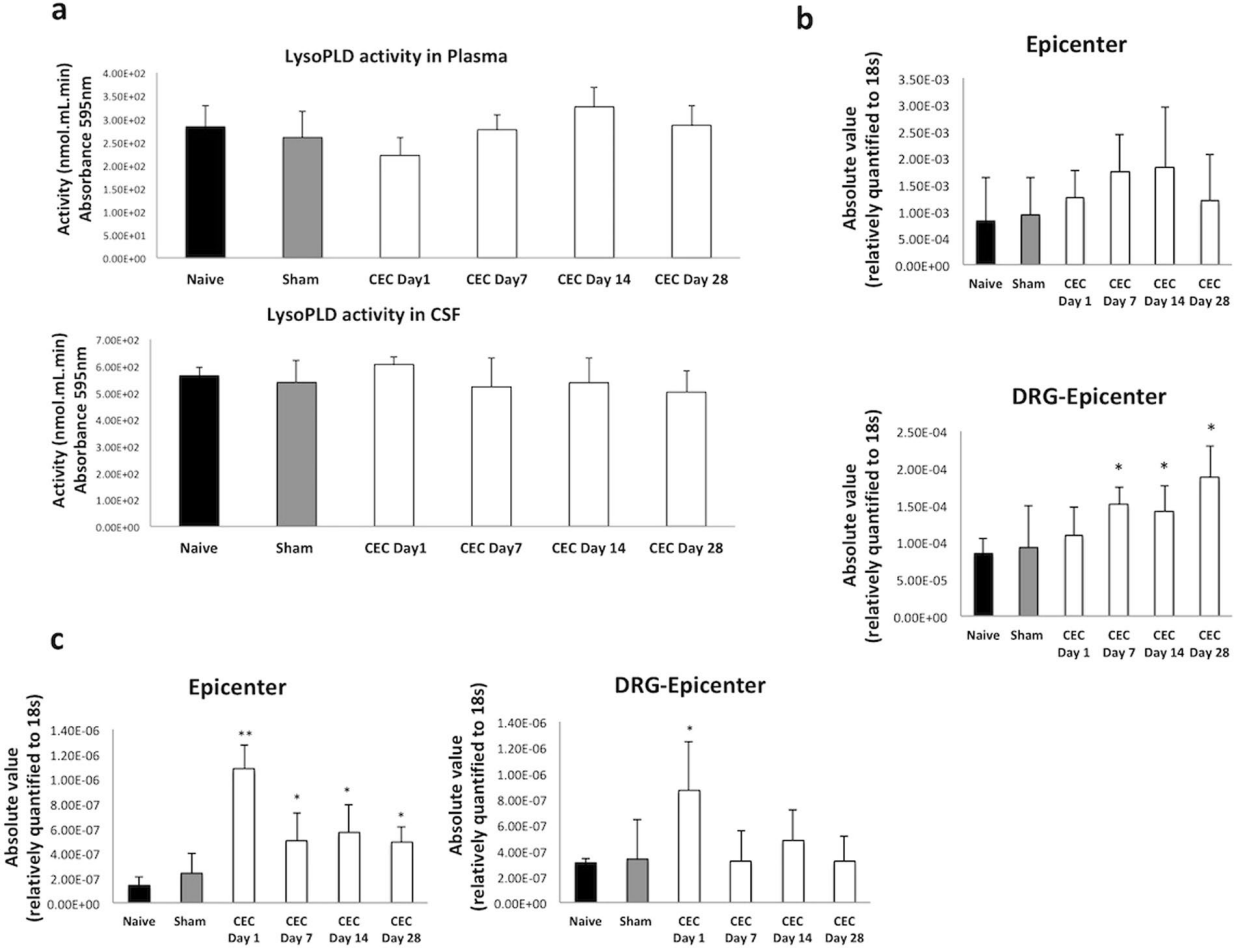
LPA synthesis pathways related factors. (**a**) LysoPLD activity was measured in the plasma and CSF of the CEC model group and compared to that of the naive and sham-operated groups. The activity in the plasma and CSF (nmol/mL/min) was not changed due to the development of LSS. (**b**) The mRNA expression levels of ATX in the spinal cord (upper) and DRGs (lower) at the epicenter segments in the CEC model group. In the spinal cord, there were no significant changes, but the expression levels in the DRGs of the CEC model group gradually increased compared to those in the naive or sham-operated group beginning on day 7. (**c**) In the epicenter segments of the spinal cord, PA-PLA1 level significantly (*p < 0.05, **p < 0.01) increased in the CEC model group compared to the naive and sham-operated groups. In the DRGs from the epicenter segments PA-PLA1 expression enhanced only at day 1 in CEC model group (*p < 0.05). We evaluated correlation between the CSF levels of measured LPA species and the corresponding LPC species, (*p < 0.05, **p < 0.01, ***p < 0.001).

Next, we evaluated the activity of ATX (ectonucleotide pyrophosphatase/phosphodiesterase 2, NPP2 or ENPP2), an LPA-producing enzyme, in the plasma and CSF as well as the mRNA expression level of ATX in the spinal cord and DRGs of the CEC model rats. As shown in Fig. 7a, high LysoPLD activity in the plasma and CSF was detected, but its level did not change between naive, sham-operated and different time points of CEC model groups, which was similar to our recent study in LSS patients^15^. The mRNA expression level of ATX was also not changed in the epicenter segments of the spinal cord but was increased in the DRGs at the spinal level of the epicenter segments compared to that in the naive and sham-operated rats beginning on day 7 after the surgical procedure (Fig. 7b).

Relating other LPLs, in plasma LPE (16:0, 16:1, 18:0, 18:1, 18:2, 18:3, 20:3, 20:4, 20:5, 22:5, 22:6), LPG (16:0, 16:1, 18:0, 18:1, 18:2, 20:3, 20:4, 22:5, 22:6), LPS (18:0, 18:1, 18:2, 20:4, 22:6) and LPI (16:0, 16:1, 18:0, 18:1, 18:2, 20:3, 20:4, 20:5, 22:5, 22:6) were measured without any significant changes in comparison to naive or sham-operated groups (data not shown). Similarly, without significant changes between different time points and naive or sham-operated groups, in CSF LPE (16:0, 18:0, 18:1, 22:6), LPG (16:0, 18:1, 18:2), LPS (18:0, 18:1, 20:4) and LPI (16:0, 18:0, 20:4) were measured (data not shown).

As for the second pathway, as described in Fig. 7c we measured mRNA expression levels of the PA-PLA1 in epicenter segment of the SC and in DRGs from epicenter. In SC tissues in comparison to naive or sham-operated group mRNA levels were significantly (*p < 0.05) increased, especially at day 1 (**p < 0.01). In DRG-epicenter, just at day 1 significant enhancement was observed. There were no changes in PA-PLA1 mRNA expression levels in the rostral segment (data not shown).

Taken together these results suggested that the increased levels of LPA in CSF might result from two possible pathways; the increased levels of LPC, a precursor of LPA, lead to LPA production extracellularly, through the action of ATX which are abundant in CSF and PA-PLA1 in neural cells might facilitate the intracellular generation of LPA.

## Discussion

The present study showed the possible involvement of lysophospholipids (LPLs), especially the ATX-LPA axis, in neurogenic claudication associated with neuropathic pain of the development of lumbar spinal canal stenosis (LSS). Using a cauda equina compression (CEC) model, we confirmed that the ATX-LPA axis is enhanced not only in the compressed spinal cord but also in the CSF and plasma. In this CEC model, similar to LSS patients we observed increased LPC and LPA in CSF and this could be an appropriate model to study ATX-LPA axis of the LSS model with neurogenic claudication. Furthermore, we observed by MALDI-MSI that signals corresponding to LPC, which is hydrolyzed to form LPA, were increased in the compressed spinal cord. These results indicate that locally produced LPC converted to LPA by ATX, and that the LPA might lead to neurogenic claudication associated with neuropathic pain through LPA receptor signaling in both the spinal cord and dorsal root ganglia (DRGs).

In the CEC model, we measured the mRNA expression levels of 6 LPA receptor subtypes (LPA_1-6_) in the spinal cord and DRGs and revealed differences in the mRNA expression patterns of the LPA receptor subtypes. In the spinal cord tissue samples, LPA_1_ mRNA was the most abundant. In the DRGs, LPA_1_ and LPA_5_ were expressed at high levels. Previous studies have shown that high expression levels of LPA_1_ in the spinal cord are associated with nerve injury and neuroinflammation^22,23^. Our results are consistent with these findings. In the spinal cord segments corresponding to the epicenter of compression, the expression of LPA_1_, LPA_5_, and LPA_6_ gradually increased (Fig. 3a), and these trends were not observed in the rostral segments of the compressed spinal cord (Supplemental Fig. 1). Although previous studies using animal models of peripheral nerve injury-induced neuropathic pain have revealed that LPA_1_ and auxiliary LPA_3_ play a crucial role in demyelination and NP^32^, our present study using a spinal cord compression model revealed that the LPA_1_ receptor and auxiliary LPA_5_ and LPA_6_ receptors may take parts in neurogenic claudication associated with NP. The differences in the area of the nervous system that is damaged may be explained by the different LPA receptors involved. Additionally, the differences in the LPA receptors involved may explain the different characteristics of central and peripheral NP. Alternatively, differences in symptoms may explain the different LPA receptors involved, because hypoesthesia and allodynia at rest are characteristic in peripheral nerve injury but not LSS and because neurogenic claudication during walking is characteristics in LSS but not peripheral nerve injury^33^. In our present study, we could not detect obvious decrease of mechanical threshold at rest that is typical NP symptoms (i.e., allodynia and hyperalgesia). Future studies should focus on differences in symptoms between NP models.

In our CEC model, two pathological conditions were inclusively observed which are the result from acute spinal cord compression, such as lumbar disc hernia protrusion, and sustained spinal cord compression, which mimics LSS in older adults. ATF3 activation in the L5 DRG (Fig. 1c) and the infiltration of CD11b/CD45-positive cells into the site of compression in the spinal cord peaked at day 1 (Fig. 1d), indicating the acute reaction to spinal cord compression. Furthermore, we observed that these changes lasted until day 28, suggesting that both neutrophil infiltration in the early phase and macrophage/microglia infiltration in the late phase may be responsible for persistent motor dysfunction. In a previous report using an animal model of SCI, time point-independent increases in the levels of LPA species, infiltration by microglia/macrophages and demyelination were observed^26^.

Of the 12 quantified species of LPC and LPA, we observed elevations in the levels of LPC 14:0, 16:0, 18:1, 18:2, 18:3, 20:5 and LPA 16:0, 18:1, 18:2, 20:3, 20:4, 20:5, 22:6 in the plasma of the CEC model rats. Normally, there are very low levels of LPLs in the CSF, but in the CEC model rats, some species of LPC and LPA were increased but not significantly; in contrast, total LPC and total LPA levels were increased significantly beginning the first day after surgery. Additionally, we observed a strong correlation between total LPC and total LPA levels in the CSF (Fig. 4c). Although the changes in LPA levels were transient, the increased expressions of LPA receptors (LPA_1_, LPA_5_ and LPA_6_ Fig. 3a) were persistent. Therefore, the stable expression of LPA in spinal cord tissues may continuously stimulate ATX-LPA signaling and trigger sustained demyelination, motor dysfunction and NP of neurogenic claudication.

In the MALDI-MSI analysis, increased LPC 16:0 and 18:1 levels were observed in the transverse spinal cord sections from the location of compression (Fig. 6b). While the relative abundance of LPC 16:0 and 18:1 in CEC by the LC-MS/MS analysis were not different in Fig. 5, the sample was including the whole area of the spinal cord, and the local concentration of molecules was diluted. We thus examined the MALDI-MSI analysis to observe the distribution of LPCs, and concluded that those molecules were changed at the small area in the spinal cord of CEC model. Additionally, such varied results may be explained by the use of different parts of the tissue samples, indicating that LPC and LPA levels in the spinal cord tissues just below the area of compression (i.e., the superficial layers of the spinal dorsal horn), but not those in the entire damaged spinal cord, are affected by epidural compression.

In this model, motor deficiencies in the form of neurogenic claudication, which is a typical symptom of LSS associated with NP, was consistently observed. In general, LSS patients complain less of allodynia in the affected leg, and no allodynic responses of the footpad were consistently observed in this model at any time point after surgery. Persistent NP in LSS is usually due to direct nerve compression at the nerve root level^1^, and the CEC model we used here is not related to the injury of the nerve root. The clinical presentations of peripheral nerve injury- and spinal cord injury-associated neuropathic pain are quite different from those of LSS^34^. To further clarify the effect of the ATX-LPA pathway on the mechanism of neurogenic claudication associated with NP due to LSS, future studies using a model more similar to radicular nerve root type LSS and a combination of spatiotemporal lipid imaging analyses are necessary. Additionally, the analysis of transverse slices was examined once because of the technical bottleneck to cut the complete slices of the exact surface to observe the distribution of metabolites by MALDI-MSI. The conductive adhesive cryofilm for MALDI-MSI analysis was developed to obtain the whole-body section including bone and spinal cord^35^. The utility will solve the bottleneck, and clearer and reproducible results of the LPC and LPA distributions should be obtained to clarify our finding in the future study.

In conclusion, we observed decreased locomotor function in behavioral studies and increased LPA levels and mRNA expression of LPA receptors in the CSF, plasma and spinal cord tissue of the CEC model rats. Specific LPA species (16:0, 18:2, 20:4) were upregulated, which have been shown to be produced by ATX detected in the CSF, without changes on its level. LPC were detected in the local regions where compression injury was induced, and it suggested that LPA were converted by ATX from these local LPC. The results of this study were similar to data obtained from human NP patients, proposing that the present CEC model is appropriate to evaluate the role of LPA signaling in the development of neurogenic claudication and NP.

## Materials and Methods

### Animals

Adult female Sprague-Dawley rats (8–10 weeks old; 200–250 gr) were used in this study and obtained from Japan SLC (Shizuoka, Izu, Japan). The rats were housed with a 12 h light/12 h dark cycle and ad libitum access to food and water. All animal experiments were conducted in accordance with the guidelines for the Care and Use of Laboratory Animals and were approved by the Ethics Committee for Animal Experiments of the University of Tokyo (approval No. P15-100).

### Surgical procedure

The cauda equina compression (CEC) model was generated by following procedures described by Watanabe K *et al*.^36^ and Khan *et al*.^37^. Briefly, the animals were anesthetized by an intraperitoneal injection of 6.5 mg/kg pentobarbital (Kyoritsu Seiyaku, Tokyo, Japan) and the inhalation of isoflurane (Abbott, Illinois, USA) and placed in a prone position. All surgical procedures were performed under a Leica M80 surgical microscope (Leica Microsystems, Buffalo Grove, IL, USA). A skin incision was made over the spinal midline, and the paraspinal muscles were separated from the spinal processes from levels L3 to L5 to expose the ligamentum flavum. Two silicon blocks (King Works CO., Ltd., Osaka, Japan) were inserted into the epidural space at L3 and L5, and the incision was closed using a sterile needle and a disposable skin stapler (Smith & Nephew, London, UK). Sham-operated animals underwent a similar surgical procedure, but silicon blocks were not inserted. The rats were returned to their cages after being placed on a 37 °C heating blanket for 1 h.

### Behavior testing

The rats were trained on the rotarod machine (Ugo Basile, Varese, Italy) for 7 days before surgery. The rotation speed was set as follows: an initial speed of 10 rpm with a gradual acceleration to 25 rpm over the first minute. After successful training, in which the rats walked for 300 sec without falling off the rod, the rats underwent CEC surgery. The time each rat spent walking until it fell off the rotating rod was measured 3 times at 10-min intervals at the following time points: before surgery and on days 1, 7, 14, and 28 after surgery (n = 5 rats from each CEC and sham-operated). We used the CEC model as a proxy for LSS, and this rotarod behavior testing was designed with assessment of neurogenic claudication associated with NP during walking. Because LSS patients hardly complain of persistent pain, allodynia and hyperalgesia at rest^1,6^, we conducted the rotarod test.

### Immunohistochemistry

For IHC, the rat tissue samples were prepared as previously described^36^. Three rats for each group used (3–5 sections per rat). The dorsal root ganglia (DRGs) from the compressed (L3, L5) and uncompressed (above L2) levels were removed and fixed in 4% paraformaldehyde in phosphate buffer solution (Wako Laboratory Chemicals, Osaka, Japan) and dehydrated in 30% sucrose. Frozen sections were cut using a cryostat (Leica CM1850, Wetzlar, Germany) at a thickness of 10 µm and mounted on slide glasses. The sections were immunostained with a mouse monoclonal antibody against ATF3 (1:300, Santa Cruz Biotechnology, Texas, USA) as the primary antibody and with a goat anti-mouse Alexa Fluor 594 (1:500, Thermo Fisher Scientific, Massachusetts, USA) antibody as the secondary antibody, and Vectashield mounting medium (Vector Laboratories, CA, USA) was used for coverslipping. The images were taken with a BZ-X700 All-in-one Fluorescence Microscope from KEYENCE Corporation (New Jersey, USA).

### Quantitative real-time polymerase chain reaction

Total RNA from the spinal cord tissues and DRGs from the silicon-compressed and uncompressed levels were extracted using TRIZOL reagent (Invitrogen, CA, USA). n = 4 rats were used from each time point. One microgram of purified total RNA was transcribed using the SuperScriptTM First-Strand Synthesis System for RT-PCR (Roche Molecular Diagnostics, CA, USA). Quantitative real-time PCR was performed with TaqMan Universal Master Mix (Applied Biosystems by Life Technologies, CA, USA) using a 7300 Real Time PCR System (Applied Biosystems). Primers and probes for ATF3, LPA1, LPA2, LPA3, LPA4, LPA5, LPA6, ATX, PA-PLA1 and the internal control ribosomal 18 s (TaqMan Gene Expression Assays) were obtained from Applied Biosystems (Rn00563784_m1, Rn00588435_m1, Rn01420531_m1, Rn00576734_m1, Rn03037115_s1, Rn02758966_s1, Rn03415828_s1, Rn01505088_m1, Rn01405321_m1 and HS99999901_s1). The samples were incubated for 10 min at 95 °C, followed by 40 cycles at 95 °C for 15 sec and 60 °C for 1 min. The mRNA expression levels of the target genes were quantified relative to ribosomal 18 s using the 2−ΔΔCt method (Applied Biosystems, User Bulletin No 2).

### Flow cytometry analysis

On days 1, 7, 14 and 28 after surgery, the rats were anesthetized deeply (n = 4 rats), and the peripheral blood was completely removed by perfusion with PBS. The spinal cord (5 mm) of the silicon-compressed and uncompressed levels were carefully dissected from the vertebral column and mechanically dissociated in 250 U/ml collagenase (Sigma Aldrich, Missouri, USA) containing HBSS (Gibco, San Diego, CA, USA). The mixtures were incubated at 37 °C for one hour and then passed through a 70 µm nylon cell strainer (Ref 352350, Falcon, Corning, NY, USA). The cells were washed in DMEM containing 10% FBS and centrifuged at 300 × g for 5 min at 4 °C. The pellets were resuspended in 2 ml stain buffer (Cat: 554656, BD Biosciences, Franklin Lakes, NJ, USA) using tubes containing cell strainers (Ref 352235), centrifuged, incubated on ice with Fc block for 15 min and incubated on ice with fluorescent antibodies for 30 min. The samples were stained with FITC mouse IgG1 (400108 Biolegend, San Diego, CA, USA), APC Mouse IgA (562140 BD Biosciences) to control for the nonspecific binding of the antibodies in the control samples and with FITC anti-rat CD45 (202205, Biolegend) and APC Mouse anti-rat CD11b (562102 BD Biosciences) to detect macrophages/microglial cells. Before analysis, propidium iodide was added to determine cell viability. All samples were suspended and analyzed at the same flow rate and duration using an Accuri C6 Flow Cytometer (BD Biosciences). The data were analyzed using FlowJo software (FlowJo LLC, USA).

### Collection of the cerebrospinal fluid (CSF) and plasma for Lysophospholipid measurements

#### CSF collection

CSF from all rats used for different time points (n = 4 rats) was collected from the cisterna magna using surgical microscopy as previously described^38^ with minor modifications. The animals were anesthetized by inhalation with isoflurane connected to a stereotaxic device (David Kopf Instruments, Tujunga, California, USA) to which the rats were mounted. A skin incision was made, the superficial muscles were dissected using bipolar forceps (Protech International Inc., San Diego, USA), and a retractor was placed under the muscle layer to carefully separate the muscles so that the dura, which is located under the muscle layer, was not accidentally punctured. The dura was exposed using 30 G 1/2 needles (Dentronics, Tokyo, Japan) connected to a 1 ml syringe, and the CSF was collected. The collected samples (50–120 µl) were labeled and stored at −80 °C.

#### Plasma collection

Blood samples were collected from the jugular vein and treated with ethylene-diamine-tetra-acetic acid and citrate-theophylline-adenosine-dipyridamole (BD Biosciences, Tokyo, Japan). The samples were centrifuged at 2500 × g for 30 min at 4 °C, and the plasma obtained was stored at −80 °C until lysophospholipid measurement^39^ (n = 4 rats).

### Measurement of Lysophospholipids (LysoPLs) in rat CSF and plasma samples

LysoPLs were quantified using LC-MS/MS, as previously described^31^. Briefly, the CSF and plasma samples and an internal standard (1 μM LPA 17:0 or 10 μM LPC 17:0) were mixed with methanol and sonicated. After centrifugation at 21,500 × g, the resulting supernatant was transferred to a sample tube for LC-MS/MS analysis. Then, the methanol extract (20 μL) was subjected to a Nanospace LC autosampler (Shiseido) equipped with a C18 CAPCELL PAK ACR column (1.5 × 250 mm; Shiseido), and LysoPLs were extracted by a gradient of solvent A (5 mM ammonium formate in water) and solvent B [5 mM ammonium formate in 95% (v/v) acetonitrile]. The eluate was sequentially ionized by ESI using a Quantum Ultra triple quadrupole mass spectrometer (Thermo Fisher Scientific). For each LysoPL class, 12 acyl chains (14:0, 16:0, 16:1, 18:0, 18:1, 18:2, 18:3, 20:3, 20:4, 20:5, 22:5, and 22:6) were monitored in both positive and negative ion modes. The concentrations of the LysoPLs were calculated based on the area ratio of the internal standard: LPA 17:0 (for LPA, LPE, LPI, LPG, and LysoPS species) or LysoPC 17:0 (for LPC species).

### Measurement of Lysophospholipids (LysoPLs) in rat spinal cord and DRG samples by UHPLC-MS/MS

The rat spinal cord was homogenized in methanol containing 0.1% formic acid (400 µL, including IS: LPA 17:0, 100 nM, LPC 17:0, 0.25 mg/mL) using a Precellys bead beater (2.8-mm zirconium oxide beads, 2 pieces, 5.000 rpm, 20 sec). After sonication for 10 min, the samples were centrifuged at 16.400 × *g* for 20 min at 4 °C, and the supernatants (300 μL) were transferred to siliconized tubes (2.0 mL). The samples were transferred to tubes (250 μL), and the samples (10 μL) were subjected to LC-MS/MS.

The LC-MS/MS analysis was performed with a NANOSPACE SI-II system equipped with dual pumps, an autosampler, and a column oven (Osaka Soda, Osaka, Japan) interfaced to a TSQ Quantiva system equipped with heated ESI interface operating in positive and negative ion mode. LysoPL analyses were performed in the selected reaction monitoring (SRM) mode. The precursor ions of LPC and LPA were [M + H]^+^ for the positive ion mode and [M − H]^−^ for the negative ion mode. The optimal product ions and collision energies are *m/z* 184.0 and 25 eV (positive ion mode) for LPC and *m/z* 153.0 and 20 eV (negative ion scanning mode) for LPA. The spray voltages in the positive and negative ion modes were 3.5 kV and 3.0 kV, respectively. The sheath gas, auxiliary gas, sweep gas, ion transfer tube temperature and vaporizer temperature were set to 45 psi, 15 psi, 2 psi, 350 °C and 400 °C, respectively. Both the sheath gas and auxiliary gas were nitrogen. The collision gas was argon at a pressure of 1.5 mTorr. The LC-MS/MS system was controlled by Xcalibur software (Thermo Fisher Scientific, San Jose, CA), and the data were collected with the same software. LC separation was performed using a reverse-phase column (Capcell Pak C8 DD, 150 mm × 1.5 mm i.d., 3 µm particle size; Osaka Soda) with a gradient elution of solvent A (5 mM ammonium formate in water) and solvent B (5 mM ammonium formate in 95% (v/v) acetonitrile) at 0.2 mL/min. The initial condition was set at 5% B. The following solvent gradient was applied: 5% B for 2 min and followed by a linear gradient to 60% B from minutes 2 to 4, 90% B from minutes 4 to 7, 100% B from minutes 7 to 10 and 100% B for 2 min. Subsequently, the mobile phase was immediately returned to the initial conditions and maintained for 4 min until the end of the run. The oven temperature was 40 °C. (n = 4 rats from each time point).

### Mass spectrometry imaging

At the selected time points, the animals were euthanized under deep anesthesia with pentobarbital (65 mg/kg, n = 3 rats). The spinal cord from levels L1 to L6 and the DRGs were freed, placed in aluminum sheets to keep the cord straight, inserted into a tube, gradually frozen in liquid nitrogen, and stored at −80 °C until MALDI-MSI analysis.

Transverse sections (8 μm) of the spinal cord were obtained with a cryostat (CM 3050 S; Leica Microsystems, Wetzlar, Germany), and coronal sections were obtained from three parts of the spinal cord, which was cut before sectioning. The sections were set on indium-tin oxide glass slides (100 ohm/sq; Matsunami, Osaka, Japan), and the glass slides were then placed in plastic tubes (50 μL) with silica gel. Then, the regions of interest of the spinal cord were exposed to laser irradiation and identified by light microscopic observation. Then, α-cyano-4-hydroxycinnamic acid (CHCA, 660 mg) was deposited on the glass slide at a thickness of 0.7 μm in an iMLayer (Shimadzu, Kyoto, Japan). The samples were immediately analyzed with MALDI-MSI (iMScope, Shimadzu).

The mass spectra of the designated areas of the spinal cord photographed before matrix application were acquired in the positive ion scanning mode and ranged from *m/z* 470 to 700. The laser irradiation time, laser power, laser irradiation diameter, laser frequency, detection voltage, and sample voltage of MALDI-MSI were 100 shots, 48, 50 μm, 1000 Hz, 1.95 kV and 3.5 kV, respectively. The data collected with the microscope system were digitally processed with imaging MS solution analysis software (Shimadzu). The software was used to merge the images of three coronal sections, and the data were normalized to the absolute intensity. The sections were stained with hematoxylin and eosin following previously described methods^40^.

### Measurement of LysoPLD activity in rat CSF and plasma samples

LysoPLD activity was assessed based on the amount of choline released with the use of LPC as the substrate, as previously described^41^. The reactions were performed in 100-µL aliquots; the serum samples (20 µL) and CSF (10 µL) were incubated with 2 mM 1-myristoyl (14:0)-LPC (Avanti Polar Lipids Inc., Alabaster, AL) in the presence of 100 mM Tris-HCl, pH 9.0, 500 mM NaCl, 5 mM MgCl_2_, 5 mM CaCl_2_, and 0.05% Triton X-100 for 3 h at 37 °C. The liberated choline was detected by an enzymatic photometric method using choline oxidase (Asahi Chemical, Tokyo, Japan), horseradish peroxidase (Toyobo, Osaka, Japan) and the TOOS reagent (N-ethyl-N-(2-hydroxy-3-sulfopropyl)-3-methylaniline; Dojindo Molecular Technologies, Inc. Tokyo, Japan) as a hydrogen donor. The choline measurement was performed with an absorption spectrometer (Infinite F50, TECAN, Zurich, Switzerland).

### Statistical analysis

Data processing and analysis were performed using R statistic software version 3.3.1 (http://www.r-project.org). Walking duration was analyzed by repeated measures ANOVA. A paired t-test was used to analyze the differences in the mRNA levels of all measured markers and in the levels of LPL species in the CSF, plasma, and tissue samples of the spinal cord and DRGs. The levels of LPL species are expressed as the means and standard deviation (SD). The statistical calculation made versus both naive and sham-operated groups. The results were considered significant when the P-value was <0.05.

## Supplementary information

Supplementary information

## References

[1] Siebert E (2009). Lumbar spinal stenosis: syndrome, diagnostics and treatment. Nat Rev Neurol.

[2] Lurie JD, Birkmeyer NJ, Weinstein JN (2003). Rates of advanced spinal imaging and spine surgery. Spine (Phila Pa 1976).

[3] Tsuda M, Inoue K, Salter MW (2005). Neuropathic pain and spinal microglia: a big problem from molecules in “small” glia. Trends Neurosci.

[4] Maier C (2010). Quantitative sensory testing in the German Research Network on Neuropathic Pain (DFNS): somatosensory abnormalities in 1236 patients with different neuropathic pain syndromes. Pain.

[5] Loeser JD, Treede RD (2008). The Kyoto protocol of IASP Basic Pain Terminology. Pain.

[6] Messiah S, Tharian AR, Candido KD, Knezevic NN (2019). Neurogenic Claudication: a Review of Current Understanding and Treatment Options. Curr Pain Headache Rep.

[7] Yaksi A, Ozgonenel L, Ozgonenel B (2007). The efficiency of gabapentin therapy in patients with lumbar spinal stenosis. Spine (Phila Pa 1976).

[8] Kasimcan O, Kaptan H (2010). Efficacy of gabapentin for radiculopathy caused by lumbar spinal stenosis and lumbar disk hernia. Neurologia medico-chirurgica.

[9] Bansal S (2016). Membrane-Stabilizing Agents Improve Quality-of-Life Outcomes for Patients with Lumbar Stenosis. Global spine journal.

[10] Orita S (2016). Pregabalin for Refractory Radicular Leg Pain due to Lumbar Spinal Stenosis: A Preliminary Prospective Study. Pain Research and Management.

[11] Dray A (2008). Neuropathic pain: emerging treatments. British journal of anaesthesia.

[12] Navarro X, Vivo M, Valero-Cabre A (2007). Neural plasticity after peripheral nerve injury and regeneration. Prog Neurobiol.

[13] Ueda H (2006). Molecular mechanisms of neuropathic pain-phenotypic switch and initiation mechanisms. Pharmacol Ther.

[14] *Wall and Melzack’s Textbook of Pain E-dition*. (Churchill Livingstone (Elsevier Health Sciences), 2005).

[15] Hayakawa K (2019). Lysophosphatidic acids and their substrate lysophospholipids in cerebrospinal fluid as objective biomarkers for evaluating the severity of lumbar spinal stenosis. Sci Rep.

[16] Aoki J, Inoue A, Okudaira S (2008). Two pathways for lysophosphatidic acid production. Biochim Biophys Acta.

[17] Mills GB, Moolenaar WH (2003). The emerging role of lysophosphatidic acid in cancer. Nature reviews. Cancer.

[18] Park KA, Vasko MR (2005). Lipid mediators of sensitivity in sensory neurons. Trends Pharmacol Sci.

[19] Saba JD (2004). Lysophospholipids in development: Miles apart and edging in. J Cell Biochem.

[20] van Meeteren LA, Moolenaar WH (2007). Regulation and biological activities of the autotaxin-LPA axis. Prog Lipid Res.

[21] Nieto-Posadas A (2011). Lysophosphatidic acid directly activates TRPV1 through a C-terminal binding site. Nat Chem Biol.

[22] David S, Lopez-Vales R, Wee Yong V (2012). Harmful and beneficial effects of inflammation after spinal cord injury: potential therapeutic implications. Handbook of clinical neurology.

[23] Choi JW, Chun J (2013). Lysophospholipids and their receptors in the central nervous system. Biochim Biophys Acta.

[24] Ueda H (2008). Peripheral mechanisms of neuropathic pain - involvement of lysophosphatidic acid receptor-mediated demyelination. Molecular pain.

[25] Ueda H (2011). Lysophosphatidic acid as the initiator of neuropathic pain. Biological & pharmaceutical bulletin.

[26] Santos-Nogueira E (2015). Activation of Lysophosphatidic Acid Receptor Type 1 Contributes to Pathophysiology of Spinal Cord Injury. J Neurosci.

[27] Kuwajima K (2018). Lysophosphatidic acid is associated with neuropathic pain intensity in humans: An exploratory study. PloS one.

[28] Nagai J, Ueda H (2011). Pre-emptive morphine treatment abolishes nerve injury-induced lysophospholipid synthesis in mass spectrometrical analysis. J Neurochem.

[29] Katz JN (2006). Lumbar disc disorders and low-back pain: socioeconomic factors and consequences. J Bone Joint Surg Am.

[30] Tsujino H (2000). Activating transcription factor 3 (ATF3) induction by axotomy in sensory and motoneurons: A novel neuronal marker of nerve injury. Mol Cell Neurosci.

[31] Kurano M (2015). Possible involvement of minor lysophospholipids in the increase in plasma lysophosphatidic acid in acute coronary syndrome. Arterioscler Thromb Vasc Biol.

[32] Ueda H (2017). Lysophosphatidic acid signaling is the definitive mechanism underlying neuropathic pain. Pain.

[33] Cobos EJ, Portillo-Salido E (2013). “Bedside-to-Bench” Behavioral Outcomes in Animal Models of Pain: Beyond the Evaluation of Reflexes. Current neuropharmacology.

[34] Behrman M, Linder R, Assadi AH, Stacey BR, Backonja MM (2007). Classification of patients with pain based on neuropathic pain symptoms: comparison of an artificial neural network against an established scoring system. European journal of pain (London, England).

[35] Saigusa D (2019). Conductive Adhesive Film Expands the Utility of Matrix-Assisted Laser Desorption/Ionization Mass Spectrometry Imaging. Analytical chemistry.

[36] Watanabe K, Konno S, Sekiguchi M, Kikuchi S (2007). Spinal stenosis: assessment of motor function, VEGF expression and angiogenesis in an experimental model in the rat. Eur Spine J.

[37] Khan M (2015). Oral administration of cytosolic PLA2 inhibitor arachidonyl trifluoromethyl ketone ameliorates cauda equina compression injury in rats. Journal of neuroinflammation.

[38] Pegg CC, He C, Stroink AR, Kattner KA, Wang CX (2010). Technique for collection of cerebrospinal fluid from the cisterna magna in rat. J Neurosci Methods.

[39] Nakamura K (2007). Suppression of lysophosphatidic acid and lysophosphatidylcholine formation in the plasma *in vitro*: proposal of a plasma sample preparation method for laboratory testing of these lipids. Analytical biochemistry.

[40] Hattori K (2010). Paradoxical ATP elevation in ischemic penumbra revealed by quantitative imaging mass spectrometry. Antioxidants & redox signaling.

[41] Umezu-Goto M (2002). Autotaxin has lysophospholipase D activity leading to tumor cell growth and motility by lysophosphatidic acid production. J Cell Biol.

