# Supplementary material for: Alteration of the lysophosphatidic acid and its precursor lysophosphatidylcholine levels in spinal cord stenosis: A study using a rat cauda equina compression model
Source: Sci Rep. 2019 Nov 12;9:16578. doi: 10.1038/s41598-019-52999-5 (PMC6851136; doi:10.1038/s41598-019-52999-5)
Supplement: Supplementary file 1 — Supplementary information [file 41598_2019_52999_MOESM1_ESM.pdf]

**Alteration of the lysophosphatidic acid and its precursor lysophosphatidylcholine in spinal cord stenosis: A study using a rat cauda equina compression model**

Uranbileg Baasanjav[1], Nobuko Ito[2]*, Makoto Kurano[1], Daisuke Saigusa[3,4], Ritsumi Saito[3,4], Akira Uruno[3,4], Kuniyuki Kano[5], Hitoshi Ikeda[1], Yoshitsugu Yamada[2], Masahiko Sumitani[6], Miho Sekiguchi[7], Junken Aoki[5] and Yutaka Yatomi[1]

**Supplemental Figure 1. Increased mRNA expression levels of LPA1, LPA5 and LPA6 in the rostral segments of the spinal cord and the DRGs following CEC.**

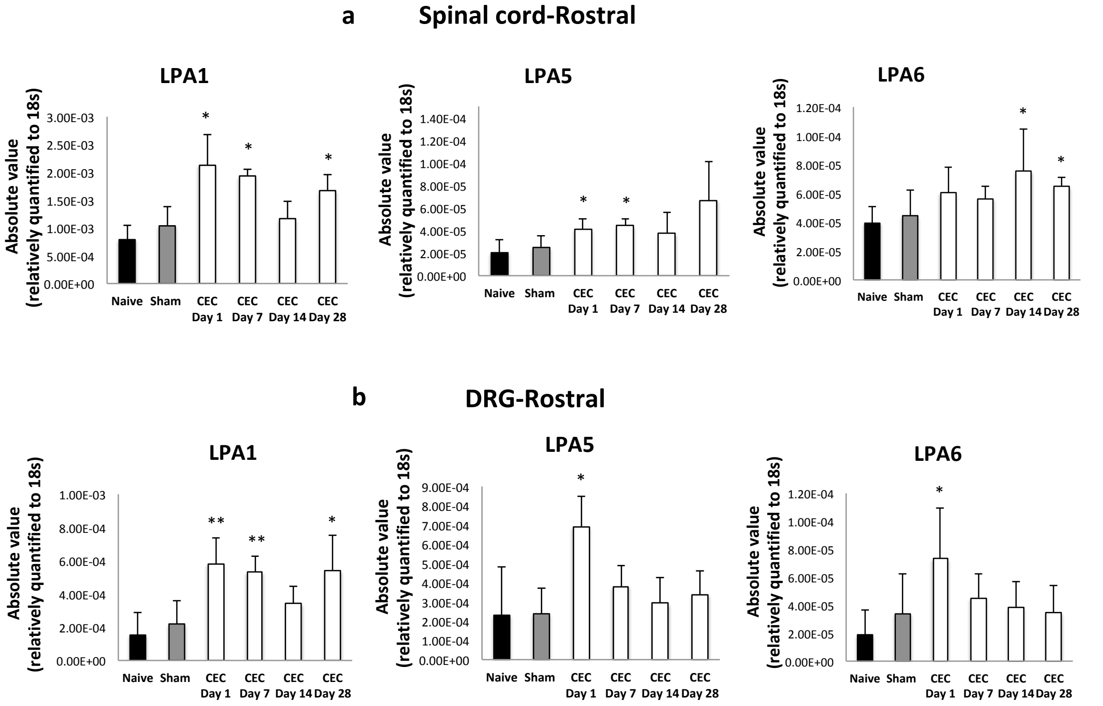


(A) In the rostral segments of the spinal cord, LPA1, LPA5 and LPA6 levels were significantly (* $p<0.05$) increased just after surgery in the CEC model group compared to the naive and sham-operated groups. A similar pattern was observed in the DRGs of the rostral segments (B) (* $p<0.05$, ** $p<0.01$).

**Supplemental Figure 2. Increased levels of LPA and LPC in the CSF and plasma of the CEC model group.**

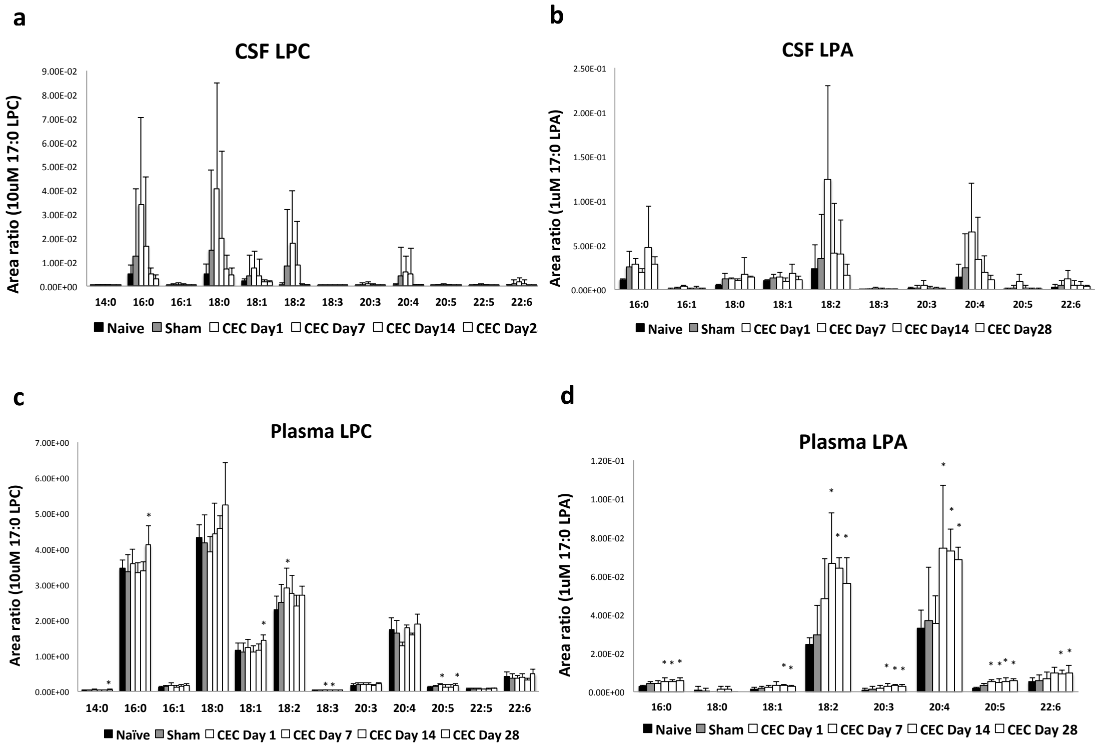


Using LC-MS/MS, LPA and LPC levels were measured in the CSF and plasma from the CEC model rats on days 1, 7, 14, 28 and from the naive, sham-operated group rat as a control. The levels of each species of LPC in the CSF (A) and in the plasma (C) were adjusted to the area ratio of 10 μM 17:0 LPC. The levels of each species of LPA in the CSF (B) and in the plasma (D) were adjusted to the area ratio of 1 μM 17:0 LPA. In the CSF the levels of both LPC and LPA were under detectable levels in the naive and sham-operated groups. However, the levels slightly increased from day 1 to day 7 in the CEC model group (A and B). None of the LPC and LPA species exhibited significantly increased levels. In the plasma, the levels of almost all LPA species were increased significantly (* $p<0.05$) in the CEC model group, whereas some LPC species were increased significantly (* $p<0.05$) (C, D).
